# Cognitive Response of Underground Car Driver Observed by Brain EEG Signals

**DOI:** 10.3390/s24237763

**Published:** 2024-12-04

**Authors:** Yizhe Zhang, Lunfeng Guo, Xiusong You, Bing Miao, Yunwang Li

**Affiliations:** 1 School of Mechanical and Electrical Engineering, China University of Mining and Technology (Beijing), Beijing 100083, China; bqt2000401001@student.cumtb.edu.cn (L.G.); bqt2100401002@student.cumtb.edu.cn (X.Y.); miaobing@student.cumtb.edu.cn (B.M.); yunwangli@cumtb.edu.cn (Y.L.); 2 Key Laboratory of Intelligent Mining Robotics, Ministry of Emergency Management, Beijing 100083, China; 3 China Academy of Safety Science and Technology, Beijing 100012, China

**Keywords:** coal mine, mine transport vehicle, driver cognition, EEG, signal processing

## Abstract

In auxiliary transportation within mines, accurately assessing the cognitive and response states of drivers is vital for ensuring safety and operational efficiency. This study investigates the effects of various vehicle interaction stimuli on the electroencephalography (EEG) signals of mine transport vehicle drivers, analyzing the cognitive and response states of drivers under different conditions to evaluate their impact on safety performance. Through experimental design, we simulate multiple scenarios encountered in real operations, including interactions with dynamic and static vehicles, personnel, and warning signs. EEG technology records brain signals during these scenarios, and data analysis reveals changes in the cognitive states and responses of drivers to different stimuli. The results indicate significant variations in EEG signals with interactions involving dynamic and static vehicles, personnel, and warning signs, reflecting shifts in the cognitive and response states of drivers. Additionally, the study examines the overall impact of different interaction objects and environments. The detailed analysis of EEG signals in different scenarios sheds light on changes in perception, attention, and responses related to drivers, which is critical for advancing safety and sustainability in mining operations.

## 1. Introduction

The cognitive load experienced by drivers is influenced by external traffic conditions and internal cognitive states, reflecting the tension and pressure felt during driving tasks or non-driving-related activities [1]. To assess these cognitive functions, electroencephalography (EEG) techniques are frequently employed [2,3]. By analyzing brain wave activity across different frequency bands, it is possible to identify various cognitive states, including rest, mental engagement, and levels of concentration or distraction [4]. Research indicates that EEG signals are highly sensitive to hazardous driving conditions, effectively distinguishing the responses of drivers to both explicit and implicit dangers [5,6].

The driving environment significantly affects the cognitive load and performance of drivers. Researchers have conducted detailed analyses of various driving scenarios, such as tunnel conditions [7,8,9], urban road conditions [10,11], and highway conditions [12,13]. In underground coal mine environments, drivers encounter unique stimuli such as narrow passages, irregular road surfaces, and specific interaction scenarios [14,15]. These factors can heighten driving risks and impact cognitive and response states. Recent research in coal mine environments has primarily focused on analyzing the concentration of remote control mine operators and monitoring the fatigue of miners. Studies have shown that various environmental and non-environmental factors can influence the concentration of remote control mine operators. Environmental factors include noise, temperature, humidity, and illumination, while non-environmental factors encompass work duration, workload, and psychological stress. These factors can elevate low-frequency EEG components and suppress high-frequency EEG components to some extent [16]. Sun et al. [17] investigate sustained attention among visual display terminal operators in smart mines by combining subjective assessment scales with objective physiological indicators. Additionally, Xu et al. [18] developed a method using a convolutional neural network (CNN) to classify the states of miners as normal, critical, or fatigued, achieving high classification accuracy by comparing EEG power spectral density and relative power spectral density. While much of the existing research focuses on monitoring work states and the impact of work environments on concentration in mining operations, there is a lack of in-depth investigation into how stimuli from mine transport vehicle interactions affect driver EEG signals. Analyzing changes in the cognitive and response states of drivers during mine transport vehicle interactions using EEG technology could provide valuable insights into brain function states and enhance driving safety and operational efficiency.

Understanding driver cognition and responses under specific interaction conditions enhances the comprehension of decision-making processes and behavioral patterns in behavioral psychology. The research results offer both empirical data and theoretical support for the fields of cognitive neuroscience and traffic psychology. The major contributions of this paper include the following:

- In a laboratory environment, we simulated various underground coal mine driving scenarios and collected real-time EEG and physiological data from multiple subjects. By utilizing eye tracking devices and EEG equipment, we recorded the eye movement patterns and brain activity of subjects while they watched videos of the mine transport vehicle. This approach ensured the accuracy and comprehensiveness of the experimental data.
- The study investigates the effects of different types of mine transport vehicle interaction stimuli, such as dynamic and static interactions, on the EEG signals of subjects.
- We employ time domain analysis, frequency domain analysis, and feature value analysis to conduct a comprehensive examination of EEG signals under various interaction stimuli experienced by subjects. Specifically, we analyze changes in EEG signals and cognitive state responses of subjects during the interaction preparation phase, the interaction process, and the recovery driving phase. This investigation reveals the neurophysiological characteristics and psychological states of drivers managing vehicle interactions in coal mine environments.

The rest of the paper is organized as follows. Section 2 presents the details of the experiment, including the experimental equipment, procedure, and data processing methods. Section 3 provides a detailed presentation of the analysis results, relevant discussions, and the general conclusions drawn from the study. Concluding remarks are presented in Section 4.

## 2. Materials and Methods

The experiment gathered real-time EEG and physiological data from multiple subjects across various driving scenarios in coal mines to investigate the impact of different types of interaction stimuli on subject EEG signals. The study focused on the EEG characteristics and their variations in response to vehicle interaction stimuli within underground coal mine environments. To achieve these objectives, a simulated driving environment was created in the laboratory using video footage captured from underground coal mine drives displayed on screens. Subjects simulated the driving process while watching these videos, with eye-tracking devices recording their gaze patterns and EEG equipment monitoring their brain activity during the video observation.

### 2.1. Experimental Platform and Equipment

**Figure 1:**
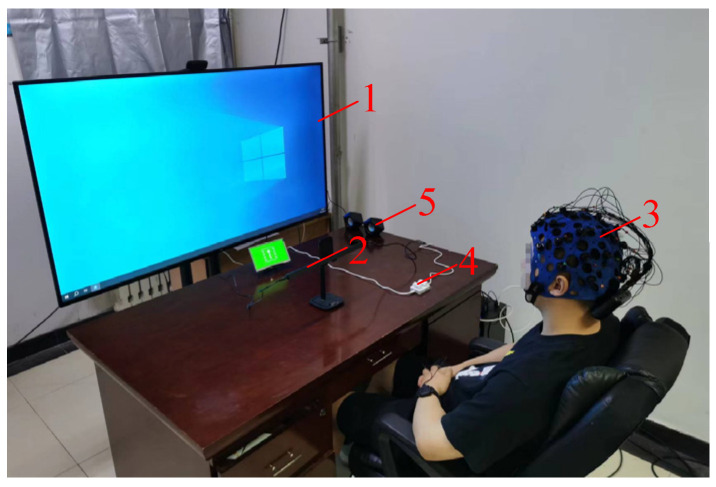
Diagram of the simulated experimental setup. 1—Driving video display screen; 2—eye tracker; 3—EEG cap; 4—button; 5—speaker.

The experimental platform utilizes the Kingfar International Inc. (Beijing, China) Research Integration System, capable of synchronously collecting eye tracking and EEG data. The simulated driving experiments were conducted in the laboratory, with the simulated experimental setup depicted in Figure 1.

The mine transport vehicle driving video was played on a 65-inch television screen connected to a Windows 10 desktop computer. The eye tracker used was the Tobii Pro Fusion remote eye tracker, with a sampling rate of 250 Hz per eye, calibrated using a five-point method. The EEG device used was the BitBrain semi-dry electrode EEG cap, with a sampling rate of 256 Hz per channel and 32 channels. Electrode placement followed the 10–20 system. During the experiment, electrode impedance was measured with pure water and maintained below 5 kΩ, with the reference electrode placed on the right earlobe. A button was used to start the driving video. Speakers were used to play ambient sounds during the driving process.

### 2.2. Stimulus Materials

**Figure 2:**
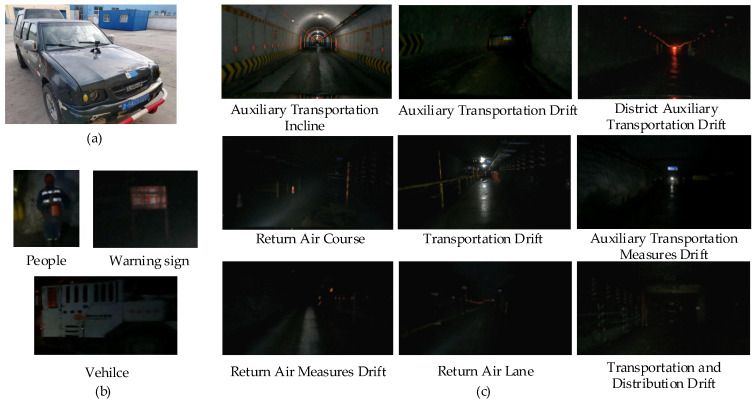
Stimulus materials. (**a**) The experimental vehicle for data collection; (**b**) types of obstacles; (**c**) types of driving video tunnels.

The experiment employed videos of mine transport vehicle operations and driving task scenarios as stimulus materials. The mine transport vehicle driving videos were recorded with a 4 K resolution camera in an underground coal mine, capturing both the visual and ambient environmental sounds during driving. The experimental vehicle is shown in Figure 2a. The types of obstacles featured include personnel, warning signs, and other vehicles, as depicted in Figure 2b. The driving videos encompassed various tunnel types, such as auxiliary transportation inclines, auxiliary transportation drifts, district auxiliary transportation drifts, return air courses, transportation drifts, auxiliary transportation measure drifts, return air measure drifts, return air lanes, and transportation measure drifts, as illustrated in Figure 2c. Tunnel scenes included straight roads, curved roads, right-angle bends, intersections, slopes, and specific conditions such as water spraying, dust, and low-light environments. A total of 36 video clips were collected, with each clip lasting between 90 and 180 s. The total duration of all videos was 1 h and 28 min.

### 2.3. Experimental Personnel

21 healthy adult volunteers participated in this experiment voluntarily. Given that all underground coal mine workers are male, only male subjects were selected. All subjects had some familiarity with coal mines, practical experience working underground, and held a valid driver’s license. To minimize individual differences, all subjects were right-handed males aged between 21 and 30 years. All subjects had no neurological or psychiatric disorders, with normal vision or corrected vision with glasses. Before the experiment, all subjects had adequate rest, and no medication, caffeine, or other stimulants were consumed, thus avoiding any factors that could potentially affect their mental state.

### 2.4. Experimental Procedure

The experiment was conducted in three sessions each day: from 9:00 a.m. to 11:00 a.m., 1:00 p.m. to 3:00 p.m., and 7:00 p.m. to 9:00 p.m. Subjects selected their preferred session time based on their individual condition and availability. Before the formal experiment began, subjects adjusted their seats to a comfortable height to minimize discomfort and distractions. All lights in the laboratory environment were turned off, and blackout curtains were used to cover all windows, ensuring that the experimental environment was completely dark and eliminating the influence of external light sources. The temperature and humidity in the room were kept constant through air conditioning. Different driving scenarios were simulated by varying the brightness on the display to represent different lighting conditions, ensuring that the brightness in the experiment matched the lighting of various driving scenarios in the underground coal mine. All subjects watched the videos under the same conditions, thereby avoiding potential biases caused by changes in ambient lighting. A 5 min baseline EEG data collection was conducted for each subject before the experiment began. During this period, all subjects were instructed to keep their eyes closed, sit quietly, and relax, ensuring that the collected data reflected the subject’s natural state without any external stimuli. The experiment began with instructional prompts, and once baseline data collection was complete and subjects were prepared, they started the experiment by pressing a button. Videos were played sequentially, followed by a rest screen during which subjects could close their eyes and rest, ensuring a stable head position to enhance the quality of subsequent experimental results. After sufficient rest, subjects resumed the experiment by pressing a button.

**Figure 3:**
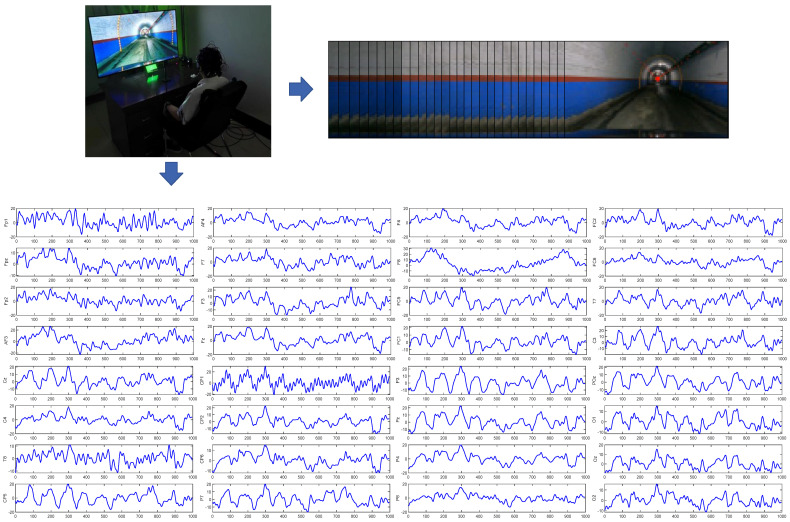
Physical diagram of data collection.

The data collection setup is illustrated in Figure 3. Each subject wore an EEG cap and was seated in front of an eye tracker. A video of a mine transport vehicle was shown on the monitor, with ambient sounds played through speakers. During the experiment, subjects were instructed to relax and watch the driving video as they would in a typical driving situation. Eye movement and EEG signals were recorded using the eye tracker and EEG equipment. In subsequent experimental analysis, combining the eye-tracking data that recorded subjects’ visual attention areas in different driving scenarios allowed for the analysis of how their visual focus changes dynamically over time. This provided contextual information for analyzing EEG signals and revealed how specific visual stimuli influenced participants’ cognitive and response states.

### 2.5. Data Processing

The collected EEG data underwent artifact removal, addressing issues such as eye blinks, eye movements, powerline interference, and noise. This was achieved using bandpass filters ranging from 1 to 49 Hz and a notch filter at 50 Hz. Subsequently, the EEGLAB toolbox was employed to apply a zero-phase high pass filter at 1 Hz and a low pass filter at 30 Hz. Next, electrode localization was performed, and a whole-head average reference was applied. Independent Component Analysis (ICA) was used to identify and remove artifacts, followed by baseline correction.

For time domain analysis, the processed EEG signals were segmented according to the experimental conditions. Subsequently, for all subjects, we employed ensemble averaging and plotted corresponding topographical maps for analysis.

For frequency domain analysis, we used the Welch [19] method to transform the time domain data into frequency domain data. Initially, the length *N* data *x*(*n*), *n* = 0, 1,…, *N* – 1 were divided into *L* segments, each containing M data points. The *i*-th segment of data is denoted as:

(1) $$x_{i}(n)=x(n+iM-M),0\leq n\leq M,1\leq i\leq L.$$

Next, we chose the Hanning window function as the window function *w*(*n*) and applied it to each signal segment. The Hanning window helps reduce spectral leakage and is suitable for signal processing where improved spectral accuracy and reduced noise interference are required. A periodogram was generated for each signal segment, denoted as:

(2) $$I_{i}(w)=\frac{1}{U}{\left|\sum_{n=0}^{M-1}x_{i}(n)w(n)e^{-jwn}\right|}^{2},i=1,2,\ldots ,M-1.$$

Here, *U* represents a normalization factor,

(3) $$U=\frac{1}{M}\sum_{n=0}^{M-1}w^{2}(n).$$

Assuming the periodograms of each segment are approximately uncorrelated, the final power spectral density estimate was computed as:

(4) $$P_{xx}(e^{jw})=\frac{1}{L}\sum_{i=1}^{L}I_{t}(w).$$

For feature value analysis, during driving, the (θ + α)/β ratio of EEG power was used to assess driver alertness. Research indicates that a lower (θ + α)/β ratio is associated with increased alertness in response to external stimuli [20,21]. To reduce individual differences and address variations in EEG power levels, we analyzed the relative power ratio (θ + α)/β.

## 3. Results and Discussion

In this section, we analyze the effects of various vehicle interaction scenarios on the signals of subjects. These scenarios encompass dynamic interaction scenes and static interaction scenes, which are further categorized into static vehicle interactions, personnel interactions, and warning sign interactions. Each scenario is divided into before-interaction, interaction, and after-interaction phases. We also assess the statistical significance of the results and discuss the implications of the derived generalizations.

### 3.1. The Effects of Dynamic Interaction Stimuli in Underground Mine on Driver EEG Signals

**Figure 4:**
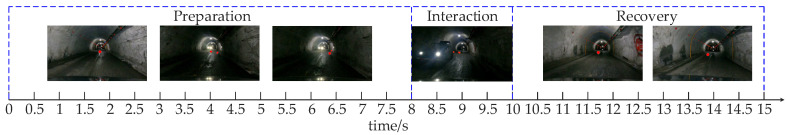
Temporal scheme of the dynamic vehicle interaction process.

In this part, we analyze dynamic vehicle interaction states, with a primary focus on time domain feature analysis, frequency domain feature analysis, and feature value analysis. The EEG signals were recorded during a 15 s dynamic vehicle interaction process, which includes preparation, interaction, and recovery stages, as shown in Figure 4.

From 1 to 8 s, the vehicle decelerates from the center of the tunnel towards the right edge in preparation for the interaction. Between 8 and 10 s, the vehicle engages in the interaction at a low speed. From 10 to 15 s, the vehicle returns to the center of the tunnel and resumes normal driving.

#### 3.1.1. Time Domain Analysis of EEG Signals Under Dynamic Vehicle Interaction Stimuli

**Figure 5:**
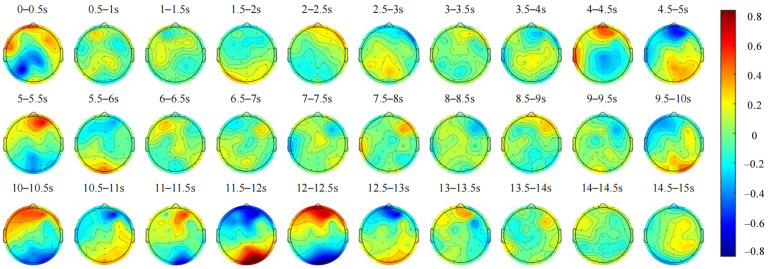
The changes over time in the average spatial topographic maps of the brain activity of all subjects during the dynamic vehicle interaction process.

The average spatial topographic maps of the EEG signals from all subjects and their changes over time are shown in Figure 5, with a 0.5 s time window representing the distribution of the average amplitude on the scalp.

Brain activity was analyzed across the frontal, parietal, occipital, and temporal lobes. Figure 5 shows that between 0.5 and 1 s, activity was notably higher in the frontal lobe. As the vehicle decelerated, activity in the frontal lobe decreased, while activity in the parietal and occipital lobes increased. Between 2 and 3 s, stimulation from oncoming vehicle headlights led to an initial increase and subsequent decrease in energy in the right frontal lobe, while energy in the parietal and occipital lobes exhibited an opposite pattern. From 4 to 6 s, as the vehicle approached the right edge of the tunnel, activity across all lobes intensified, with a marked increase observed in the AF4 electrode of the right frontal lobe. By 6 to 8 s, as the vehicle reached its interaction position, activity across all lobes decreased. During the 8 to 10 s interaction phase, energy in the right frontal lobe initially increased and then decreased, while energy in the left frontal lobe decreased. Energy in the occipital lobe initially decreased and then increased, while energy in the left temporal lobe decreased. From 10 to 13 s, as the vehicle moved back toward the center of the tunnel, activity in all lobes increased significantly. Finally, from 13 to 15 s, as the vehicle resumed normal driving, activity across all lobes decreased, with reductions in frontal and occipital lobe energies and an increase in parietal lobe energy. These observations reveal significant variations in brain activity across different regions during the dynamic vehicle interaction process, illustrating distinct temporal patterns associated with each brain region.

#### 3.1.2. Frequency Domain Analysis of EEG Signals Under Dynamic Vehicle Interaction Stimuli

EEG signals are categorized into Delta (1–4 Hz), Theta (4–8 Hz), Alpha (8–14 Hz), Beta (14–30 Hz), and Gamma (>30 Hz) waves based on their frequency, amplitude, and physiological characteristics, each corresponding to distinct cognitive functions such as cognition and thought processes. We focus on the Delta, Theta, Alpha, and Beta frequency bands, and the average power spectral density of the four frequency bands for all subjects was calculated using the Welch method. To compare the changes in energy across different frequency bands during different time periods, the data were grouped into 0.5 s intervals based on the timing of dynamic vehicle interactions. The average energy differences for each frequency band across different time intervals were calculated. The Kruskal–Wallis test was used for analysis, as it does not rely on the assumption of normality and is suitable for data with various distributions. A *p* < 0.05 was considered statistically significant [22]. Table 1 presents the results of the non-parametric test analysis of the average power for all subjects across the time interval groups for the four frequency bands, which shows significant differences in the average energy of each frequency band across different time intervals.

**Figure 6:**
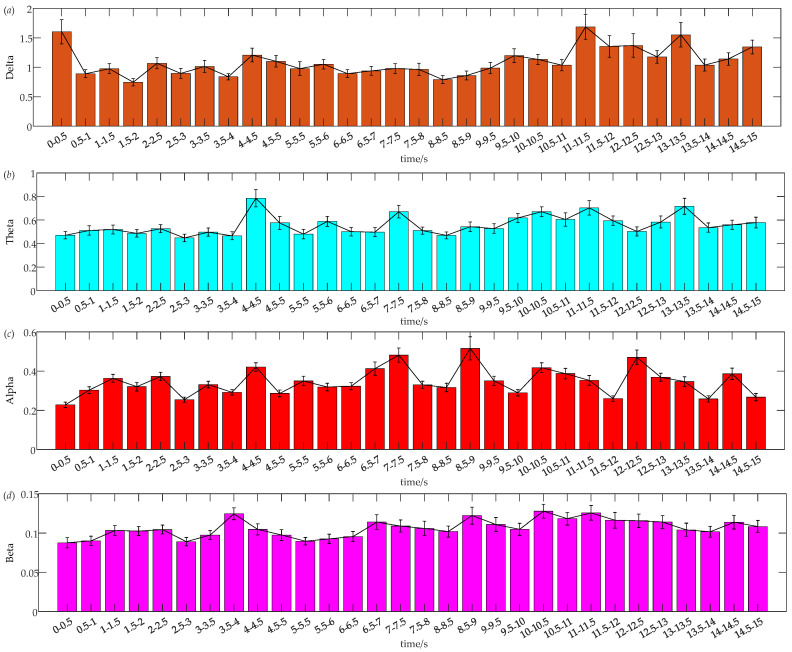
Comparison of average power across different frequency bands at different time intervals during the dynamic vehicle interaction process for all subjects. (**a**) Delta band power at different time intervals; (**b**) Theta band power at different time intervals; (**c**) Alpha band power at different time intervals; (**d**) Beta band power at different time intervals.

Figure 6 presents a bar chart showing the average differences in EEG power across four frequency bands for all subjects at different time intervals, with the data organized according to the interaction process timeline. Delta waves, the slowest brainwaves, typically occur during deep sleep and are associated with profound relaxation, recovery, and increased mental effort [23]. Figure 6a illustrates that Delta-band energy begins at a relatively high level. During the preparation and interaction phases of dynamic vehicle interaction, Delta-band energy initially declines, then rises prominently around 11.5 s, and maintains a high level thereafter. Specifically, in the preparation phase, Delta-band energy decreases within the first 2 s, slightly increases over the next 1.5 s, then declines again from 3.5 to 4 s. After 4 s, Delta-band energy continues to decrease until a slight rise at 6 s, followed by stabilization until 8 s. In the interaction phase, Delta-band energy shows a steady increase from 8 to 10 s. During the recovery phase, Delta-band energy decreases from 10 to 11 s, peaks sharply between 11 and 11.5 s, and then declines over the next 1.5 s. From 13 s onward, Delta-band energy first increases, then fluctuates, culminating in a continuous rise in the final second.

Theta waves are associated with heightened attentional engagement during specific tasks [24]. Figure 6b illustrates that Theta-band energy remains relatively low during the preparation and interaction phases of dynamic vehicle interaction, but it increases significantly during the recovery driving phase. During the preparation phase, Theta-band energy remains low for the first 4 s, increases from 4 to 4.5 s, and then decreases in the following second. Between 5.5 and 7 s, Theta-band energy initially decreases, then increases and decreases again within the subsequent second. In the interaction phase, Theta-band energy generally shows an upward trend. During the recovery phase, Theta-band energy initially decreases, then rises from 10.5 to 11.5 s, decreases continuously thereafter, briefly increases at 12.5 s, decreases again at 13.5 s, and ends with a steady rise in the final second.

Alpha waves generally appear during relaxation and closed-eye resting states, with a reduction in Alpha-band energy indicating increased attentional focus on specific tasks [24]. During driving, moderate Alpha waves can aid in maintaining alertness, whereas excessive Alpha waves may lead to lapses in driver attention. Figure 6c demonstrates notable fluctuations in Alpha-band energy. In the preparation phase of dynamic vehicle interaction, Alpha-band energy begins at a low level and rises steadily within the first 1.5 s. From 1.5 s onward, Alpha-band energy exhibits continuous fluctuations, decreasing from 5.5 to 6 s, increasing steadily over the next 1.5 s, and then decreasing again in the final 0.5 s of this phase. During the interaction phase, Alpha-band energy initially rises from 8 to 10 s and then decreases steadily. In the recovery driving phase, Alpha-band energy decreases continuously from 10 to 12 s, sharply increases from 12 to 12.5 s, decreases steadily over the next 1.5 s, rises again from 14 to 14.5 s, and finally decreases in the last 0.5 s.

Beta waves are high-frequency brainwaves commonly linked to alertness, focus, and cognitive activities. Moderate levels of Beta waves support the maintenance of alertness and attention to road conditions, while excessive Beta waves may signify increased tension or anxiety. Figure 6d depicts Beta-band energy, which demonstrates overall stability with moderate fluctuations. During the preparation phase of dynamic vehicle interaction, Beta-band energy begins at a low level, increases within the first 2 s, and then remains relatively stable. A decrease occurs from 2.5 to 3 s, followed by a continuous increase over the subsequent second. Between 4 and 7 s, Beta-band energy exhibits alternating decreases and increases, eventually settling into a consistent decline from 7 s onwards. In the interaction phase, Beta-band energy rises from 8 to 10 s and then steadily decreases. During the recovery driving phase, Beta-band energy first increases, then decreases from 10 to 11.5 s, and continues to decline over the next 2.5 s. Toward the end of this phase, Beta-band energy shows a pattern of increase followed by a decrease in the final second.

**Figure 7:**
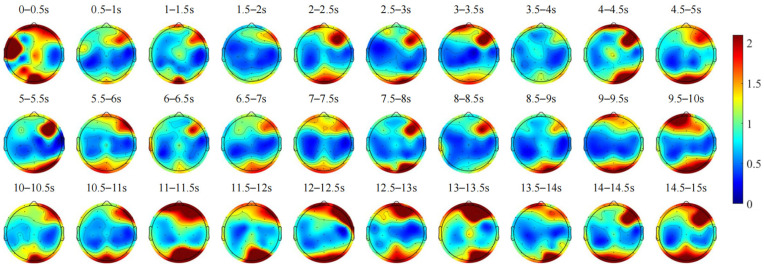
Time-varying changes in the average scalp topographic maps of Delta frequency band EEG during the dynamic vehicle interaction process for all subjects.

Figure 7 shows the changes over time in the average scalp topographic maps of the Delta frequency band for all subjects. The asymmetry observed in Delta waves within the occipital lobe may relate to visual information processing or spatial perception, suggesting atypical brain activity in these functions. Conversely, asymmetry in the frontal lobe may indicate cognitive processes related to attention, emotion, or cognitive adjustments. During the preparation phase of dynamic vehicle interaction, from 0 to 2 s, as the vehicle decelerates, Delta-band energy in the frontal and occipital lobes fluctuates, reflecting varying levels of attention to environmental cues and changes in cognitive load. Between 2 and 2.5 s, Delta-band energy increases in both lobes, likely in response to stimuli such as the flashing headlights of oncoming vehicles, which shifts attention towards the approaching vehicles. Subsequently, from 2.5 to 3 s, Delta-band energy decreases as attention focuses on the approaching vehicle. Between 3.5 and 6 s, as the vehicle approaches the right side of the tunnel, a similar fluctuating pattern in Delta-band energy is observed, indicating increased visuospatial attention. From 6.5 to 7.5 s, a slight rise in Delta-band energy in both lobes may signify the completion of interaction preparation and heightened attention to surrounding vehicle movements. Throughout the dynamic vehicle interaction phase, as vehicles interact and move out of sight, attention becomes increasingly focused on the remaining vehicle. From 8 to 8.5 s, Delta-band energy decreases in the occipital lobe and remains lower in the frontal lobe, which may reflect reduced visual processing demands as the interaction concludes. During the recovery driving phase, from 10 to 10.5 s, as the vehicle moves from the right side of the tunnel to the centerline position, Delta-band energy decreases in the frontal pole left and occipital lobes, indicating focused attention on vehicle positioning. Subsequent periods reveal sustained high Delta-band energy in both lobes, suggesting continued heightened visuospatial attention and a return to a regular driving state. Based on the above analysis, it can be concluded that during vehicle deceleration, the driver’s attention is focused on the surrounding environment, leading to changes in the Delta-wave energy in the occipital and frontal lobes. In the interaction preparation and interaction phases, the increased visual spatial attention may lead to an enhancement of Delta-wave energy. This is consistent with the findings in the literature [25]. These changes may reflect the brain’s dynamic regulation in response to complex driving tasks, particularly the demands related to visual and spatial information processing.

**Figure 8:**
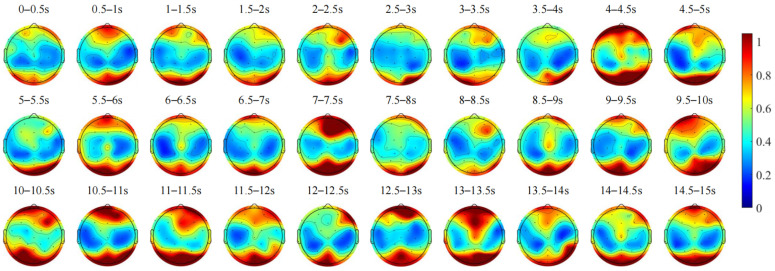
Time-varying changes in the average scalp topographic maps of Theta frequency band EEG during the dynamic vehicle interaction process for all subjects.

In Figure 8, during the preparatory phase of dynamic vehicle interaction, Theta-band energy remains notably high in the occipital lobe, while the frontal lobe exhibits fluctuating Theta-band energy, likely reflecting cognitive function maintenance and shifts in attentional states. Specifically, from 1.5 to 2 s and from 3.5 to 4 s, both lobes show decreased Theta-band energy, which suggests increased driver focus and alertness. However, from 6.5 to 7.5 s, there is an increase in Theta-band energy across the frontal, parietal, and occipital lobes, indicating potential distraction. These observations are consistent with the scalp topography analysis of Delta band activity. During the dynamic vehicle interaction phase, Theta-band energy in the frontal and occipital lobes initially decreases before increasing, reflecting fluctuations in cognitive load and attention levels during the interaction. Attention becomes more concentrated during the vehicle interaction phases, followed by a realization that the interaction can proceed smoothly with reduced attention. In the recovery driving phase, Theta-band energy remains relatively high in the frontal pole right and occipital lobes, which may indicate dispersed attention during the transition back to normal driving. Based on the above analysis, it can be observed that the enhancement of Theta-wave activity in the frontal lobe is typically associated with increased cognitive load, focused attention, and decision-making processes. Studies have shown that the frontal lobe is closely related to higher cognitive tasks such as planning, attention regulation, and decision-making [26]. In the dynamic interaction preparation phase, the increase in Theta energy in the frontal lobe can be interpreted as the driver entering a high-attention state, preparing for perception and decision-making. The occipital lobe, primarily responsible for visual processing, shows increased Theta-wave energy, which may be related to the driver’s heightened attention to the surrounding environment, particularly visual information. This is consistent with the key role of the occipital lobe in visual information processing.

**Figure 9:**
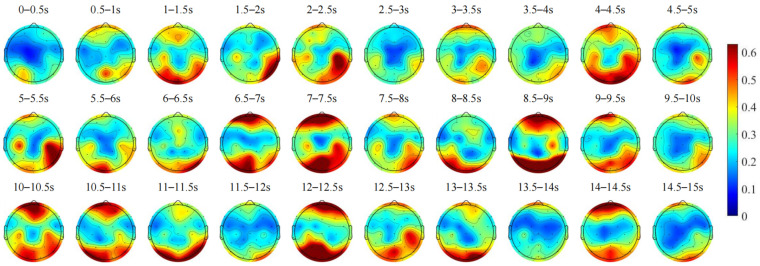
Time-varying changes in the average scalp topographic maps of Alpha frequency band EEG during the dynamic vehicle interaction process for all subjects.

Alpha waves reflect the state of mental relaxation, with stronger Alpha-band energy indicating greater relaxation. In Figure 9, during the preparatory phase of dynamic vehicle interaction, Alpha-band energy in the frontal and occipital lobes initially increases and then decreases from 0 to 2 s. This pattern likely reflects the initial tension experienced by subjects, which diminishes as the vehicle decelerates and spatial attention increases. However, from 1.5 to 2 s, increased focus on specific areas may elevate tension, resulting in decreased Alpha energy in these lobes. From 2 to 2.5 s, a surge in Alpha-band energy may occur in response to stimuli such as flashing headlights from oncoming vehicles, prompting a shift in visual attention. In the following 0.5 s, heightened focus on the oncoming vehicle increases tension, leading to decreased Alpha-band energy in both lobes, consistent with changes observed in Delta and Theta bands during corresponding periods. From 6.5 to 7.5 s, an increase in Alpha-band energy in the frontal and occipital lobes may indicate the completion of interaction preparation and a transition to a more relaxed state. During the dynamic vehicle interaction phase, from 8 to 8.5 s, decreased Alpha energy in the frontal lobe suggests focused attention, while from 8.5 to 9 s, increased Alpha-band energy in both lobes may reflect brief relaxation upon realizing successful interaction. Between 9 and 10 s, decreased Alpha-band energy in these lobes suggests heightened tension as brain resources are reallocated after completing interaction tasks, preparing for subsequent cognitive control and decision-making processes. In the recovery driving phase, from 10 to 12 s, a gradual decrease in Alpha-band energy in the frontal and occipital lobes may indicate increased tension as the vehicle resumes normal driving. Subsequent increases in energy levels may signify a relatively relaxed state during routine driving without external stimuli, with attention once again diminishing. Low Alpha-wave energy is associated with increased cognitive activity, attention, and information processing. This is consistent with the conclusions in the literature [27], which suggest that increased attention and task pressure reduce Alpha-wave energy, while Alpha-wave energy rises when the task is completed or attention shifts. Throughout different phases of the driving task, changes in the driver’s psychological stress and emotional state are closely related to fluctuations in Alpha-wave activity. In stressful driving environments, Alpha-wave energy decreases, while in relaxed states, Alpha-wave energy increases.

**Figure 10:**
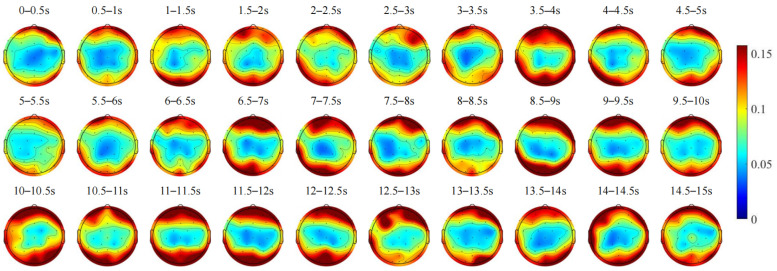
Time-varying changes in the average scalp topographic maps of Beta frequency band EEG during the dynamic vehicle interaction process for all subjects.

Beta-band energy is associated with sensitivity to external stimuli and alertness. In Figure 10, Beta-band energy remains high throughout the entire process, indicating sustained heightened alertness while driving through the tunnel.

#### 3.1.3. Characteristics Analysis of EEG Signals Under Dynamic Vehicle Interaction Stimuli

**Figure 11:**
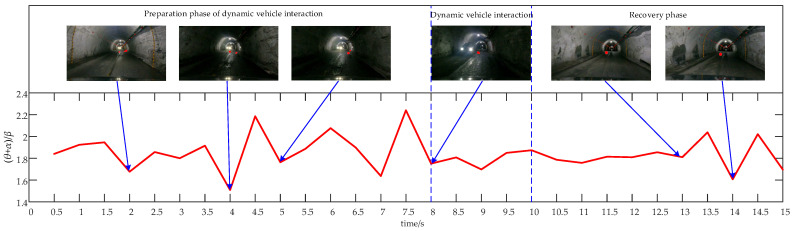
Time-course of the average relative power combination values of (θ + α)/β across all brain regions for all subjects during the dynamic vehicle interaction process.

Figure 11 illustrates the average relative (θ + α)/β power values for all subjects across different stages of dynamic vehicle interaction. The average relative power ratio (θ + α)/β throughout this process ranges from 1.51 to 2.24, reflecting consistently high levels of alertness. During the preparation phase, drivers must quickly identify an appropriate interaction position. At this stage, the vehicle approaches the tunnel edge, slows to the interaction speed, and encounters stimuli such as flashing lights from other vehicles, which heightens driver alertness. Alertness decreases after completing the preparation but increases again as vehicles approach. In the dynamic vehicle interaction phase, drivers engage at low speeds after having previously identified the interaction position, which facilitates smooth interaction and leads to a decrease in alertness. Following the interaction, the vehicle accelerates from the right side of the tunnel towards the centerline in the absence of other vehicles, resulting in reduced driver alertness. As the vehicle reaches the middle of the tunnel and normal driving conditions are restored, alertness increases once more.

### 3.2. The Effects of Static Interaction Stimuli in Underground Mine on Driver EEG Signals

This section utilizes feature value analysis to examine typical static object interaction states in underground coal mines, including interactions with static vehicles, personnel, and warning signs.

#### 3.2.1. Characteristics Analysis of EEG Signals Under Static Vehicle Interaction Stimuli

**Figure 12:**
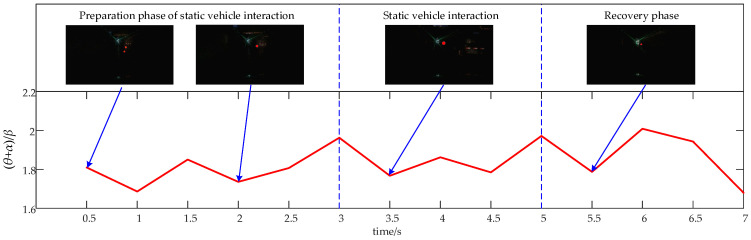
Time-course of the average relative power combination values of (θ + α)/β across all brain regions for all subjects during the static vehicle interaction process.

EEG signals recorded during a 10 s static vehicle interaction process are divided into preparation (0–3 s), interaction (3–5 s), and recovery stages (5–7 s). Between 1 and 3 s, the driver detects a parked vehicle on the left side of the tunnel. The interaction with the vehicle occurs from 3 to 7 s, after which the vehicle departs from its interaction position between 7 and 10 s, returning to normal driving conditions. Figure 12 illustrates the average relative (θ + α)/β power values for all subjects across different stages of static vehicle interaction.

Figure 12 illustrates that the average relative power combination value of (θ + α)/β during the static vehicle interaction process varies from 1.47 to 2.23. In the preparatory phase, the driver first observes a parked vehicle on the left edge of the tunnel, leading to increased alertness. Focus shifts primarily to the right side of the parked vehicle. As the vehicle moves closer and it becomes apparent that the parked car will not obstruct normal driving, alertness decreases. During the static vehicle interaction phase, alertness rises again as the vehicle engages with the parked car, then tapers off as the interaction ends. In the recovery phase, alertness initially increases in preparation for subsequent driving tasks, then decreases as normal driving conditions resume but remains at a level adequate for ongoing tasks.

#### 3.2.2. Characteristics Analysis of EEG Signals Under Personnel Interaction Stimuli

**Figure 13:**
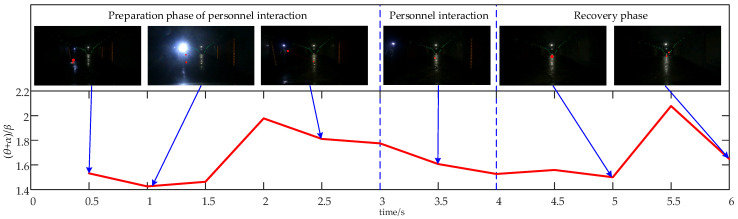
Time-course of the average relative power combination values of (θ + α)/β across all brain regions for all subjects during the personnel interaction process.

The EEG signals recorded during a 7 s personnel interaction process can be divided into three distinct stages: preparation (0–3 s), interaction (3–4 s), and recovery stages (4–6 s). During the initial 3 s, personnel positioned on the left side of the tunnel were observed, with stimuli originating from their headlamps. From 3 to 4 s, the vehicle interacts with the personnel. Following this interaction, the vehicle moves away from the personnel between 4 and 6 s, transitioning back to normal driving conditions thereafter. Figure 13 illustrates the average relative (θ + α)/β power values for all subjects across different stages of personnel interaction.

As illustrated in Figure 13, the average relative power combination value (θ + α)/β during the personnel interaction process ranges from 1.43 to 2.08, reflecting increased alertness. In the preparation phase, the driver initially focuses on individuals to the left of the tunnel, leading to a steady rise in alertness. Between 1 and 1.5 s, attention shifts upward due to stimuli from personnel headlamps, followed by alternating focus between the personnel and the forward driving direction during the next 1.5 s, which causes a slight dip in alertness. During the personnel interaction phase, alertness gradually increases with the ongoing interaction. In the recovery phase, as the vehicle returns to normal driving conditions, alertness decreases from 5 to 5.5 s but remains relatively high during other intervals.

#### 3.2.3. Characteristics Analysis of EEG Signals Under Obstacle Interaction Stimuli

**Figure 14:**
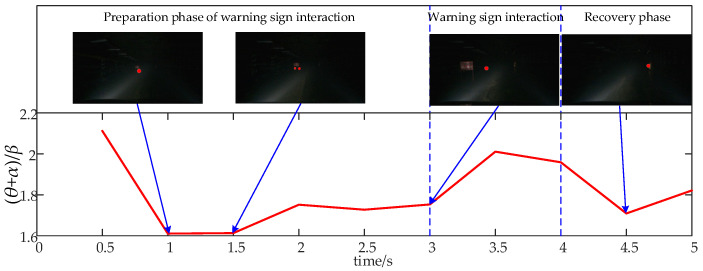
Time-course of the average relative power combination values of (θ + α)/β across all brain regions for all subjects during the obstacle interaction process.

The EEG signals recorded during the 5 s interaction with warning signs in the mine can be divided into preparation (0–3 s), interaction (3–4 s), and recovery stages (4–5 s) with the warning sign. Initially, from 1 to 3 s, the driver notices the warning sign on the left side of the tunnel while continuing to drive forward. Between 3 and 4 s, the vehicle interacts directly with the warning sign. From 4 to 5 s, the vehicle moves away from the warning sign and transitions back to normal driving mode. Figure 14 illustrates the average relative (θ + α)/β power values for all subjects across different stages of obstacle interaction.

Figure 14 shows that the average relative power combination value (θ + α)/β during the interaction with the warning sign ranges from 1.61 to 2.11, reflecting consistently high levels of alertness. Prior to interacting with the warning sign, the driver first notices it on the left side of the tunnel, which increases alertness. This heightened state of alertness continues during the interaction phase. However, as the driver recognizes that a smooth interaction is feasible, attention may briefly shift toward the direction of travel, causing a temporary decrease in alertness.

### 3.3. Different Interaction Object Stimulus Correlation Analysis

To analyze the impact of different interaction objects on the EEG signals of subjects, data from interactions involving static vehicles, personnel, and warning signs were selected. Each interaction type was divided into three phases: 1 s before interaction, 1 s during interaction, and 1 s after interaction. EEG channels were categorized into four brain regions: frontal lobe, parietal lobe, occipital lobe, and temporal lobe. The average power spectral density of δ, θ, α, and β waves was extracted for each of these brain regions.

**Figure 15:**
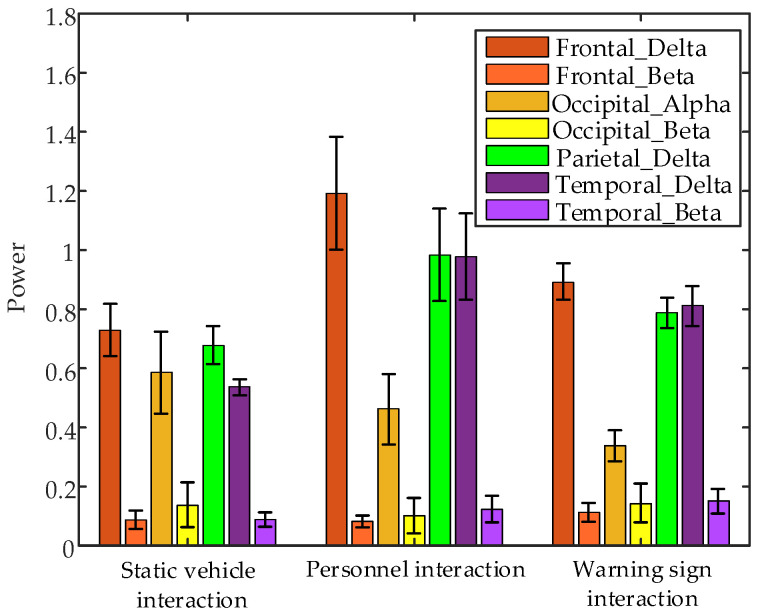
Error bar plots of the average EEG parameters of all subjects during the 1 s before interaction with different interaction objects.

Non-parametric tests [25] performed on EEG data 1 s before interactions with static vehicles, personnel, and warning signs revealed significant differences in several brain regions, specifically frontal lobe δ band power (*p* = 0.0273 < 0.05) and β band power (*p* = 0.0003 < 0.05), occipital lobe α band power (*p* = 0.0059 < 0.05) and β band power (*p* = 0.0161 < 0.05), parietal lobe δ band power (*p* = 0.0273 < 0.05), temporal lobe δ band power (*p* = 0.0390 < 0.05) and β band power (*p* = 2.3974 × 10^−5^ < 0.05) have significant differences. Figure 15 presents error bar plots of the average EEG parameters of all subjects during the 1 s before interaction with different objects. The results indicate that, in the frontal lobe, δ band power is highest during interactions with personnel, while β band power peaks during interactions with warning signs. In the occipital lobe, β band power is highest during interactions with both static vehicles and warning signs. In the parietal lobe, α band power is highest during interactions with personnel. In the temporal lobe, δ band power is highest during interactions with personnel, whereas β band power is highest during interactions with warning signs.

**Figure 16:**
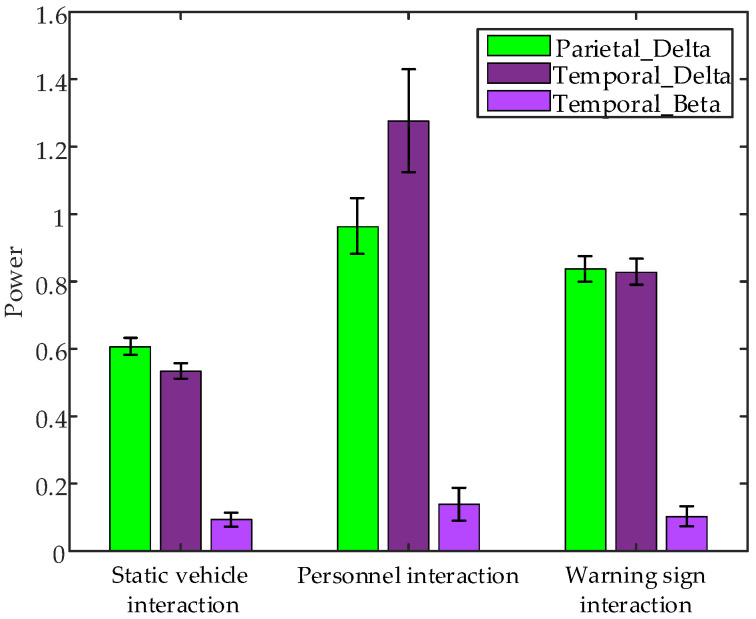
Error bar plots of the average EEG parameters of all subjects during the 1 s interaction with different interaction objects.

Non-parametric tests conducted on EEG data collected during 1 s of interaction with static vehicles, personnel, and warning signs revealed significant differences in several brain regions, specifically parietal lobe δ band power (*p* = 0.0390 < 0.05), temporal lobe δ band power (*p* = 0.0273 < 0.01) and temporal lobe β band power (*p* = 0.0007 < 0.01). Figure 16 presents error bar plots of the average EEG parameters of all subjects during the 1 s interaction with different objects. The results indicate that δ band power in the parietal lobe and the δ and β band powers in the temporal lobe are highest during interactions with personnel and lowest during interactions with static vehicles.

**Figure 17:**
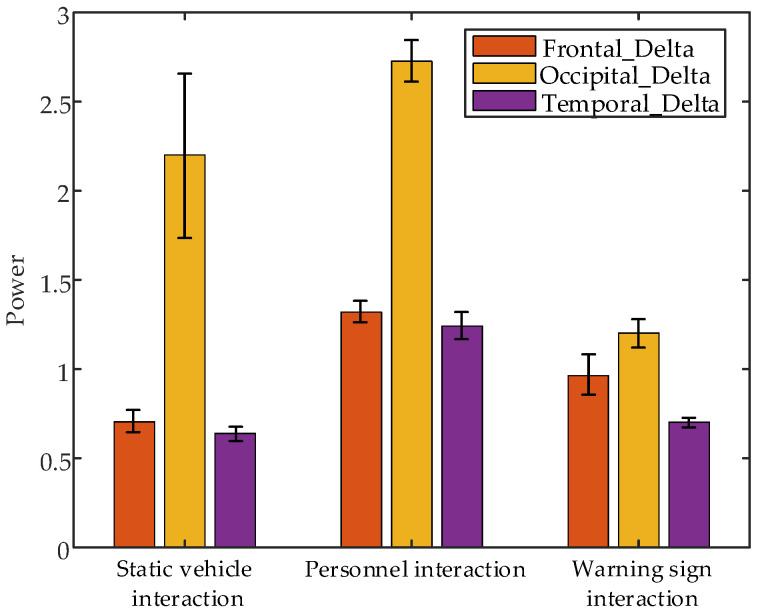
Error bar plots of the average EEG parameters of all subjects during the 1 s after interaction with different interaction objects.

Non-parametric tests conducted on EEG data collected during the first second after interactions with static vehicles, personnel, and warning signs revealed significant differences, specifically frontal lobe δ band power (*p* = 0.0273 < 0.05), occipital lobe δ band power (*p* = 0.0273 < 0.05) and temporal lobe δ band power (*p* = 0.0273 < 0.05). Figure 17 presents error bar plots of the average EEG parameters of all subjects during the 1 s after interaction with different objects. The results indicate that δ band power is highest in the frontal, occipital, and temporal lobes after interactions with personnel. Conversely, δ band power is lowest in the frontal and temporal lobes after interactions with static vehicles and lowest in the occipital lobe after interactions with warning signs.

The comprehensive analysis reveals significant differences in EEG signals associated with various interaction objects during the 1 s prior to interaction, predominantly in the frontal, occipital, parietal, and temporal lobes. During the 1 s of interaction and the 1 s after interaction, fewer significant differences were observed. Specifically, notable differences during the interaction were mainly found in the parietal and temporal lobes, whereas differences observed 1 s after interaction were primarily in the frontal, occipital, and temporal lobes. EEG signals during interactions with personnel displayed more pronounced characteristics compared to interactions with static vehicles and warning signs, with notably higher power values in the delta frequency band. This may be due to increased alertness triggered by the flickering headlamps worn by personnel in underground environments, and the warning signs potentially enhancing alertness more effectively than static vehicles.

### 3.4. Different Interaction Position Stimulus Correlation Analysis

**Figure 18:**
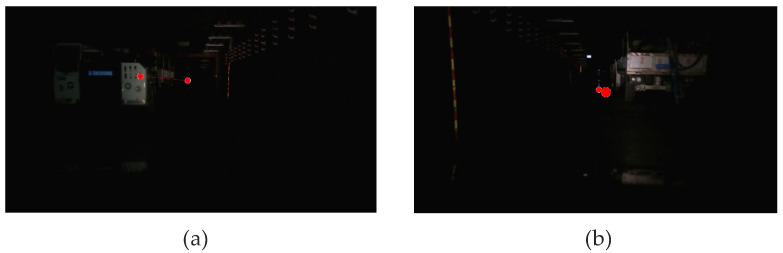
Diagram of static vehicle interaction in different position. (**a**) Interaction with a stationary vehicle on the left side; (**b**) interaction with a stationary vehicle on the right side.

To analyze the effect of different interaction positions on subject EEG signals, EEG data from interactions with static vehicles on the left and right sides were selected for examination. Data were extracted from 1 s before the interaction, during the 1 s interaction, and 1 s after the interaction. Feature selection followed the methodology outlined in Section 3.3. Figure 18 illustrates the different interaction positions.

**Figure 19:**
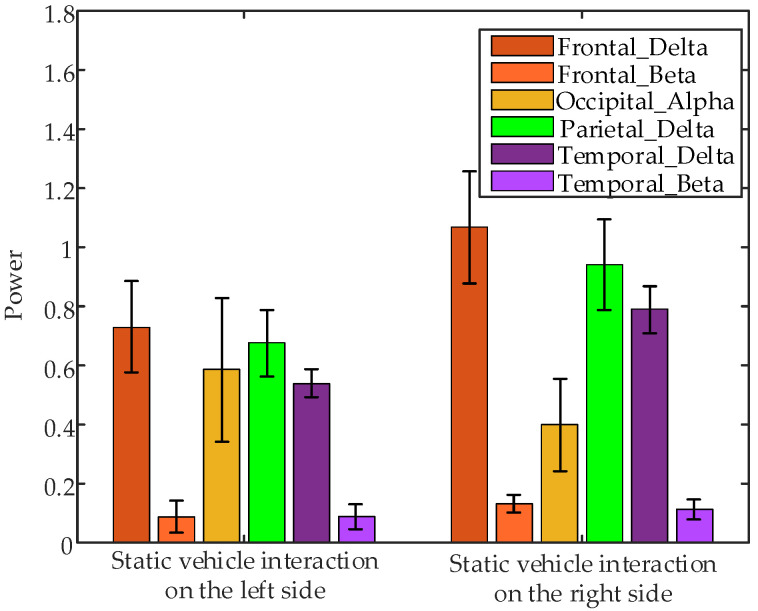
Error bar plots of the average EEG parameters of all subjects during the 1 s before interaction under different interaction positions.

Non-parametric tests on EEG data collected 1 s before interaction at different positions revealed significant differences in frontal δ band power (*p* = 0.0495 < 0.05), frontal β band power (*p* = 0.0001 < 0.05), occipital α band power (*p* = 0.0250 < 0.05), parietal δ band power (*p* = 0.0495 < 0.05), temporal δ band power (*p* = 0.0495 < 0.05), and temporal β band power (*p* = 0.0076 < 0.05) between interaction with static vehicles on the left and right sides. Figure 19 shows the error bar plots of the average EEG parameters of all subjects during the 1 s before interaction at different positions. The results indicate that only the occipital α band power was higher for interactions with static vehicles on the left side compared to the right side, while other significantly different features were lower on the left side.

**Figure 20:**
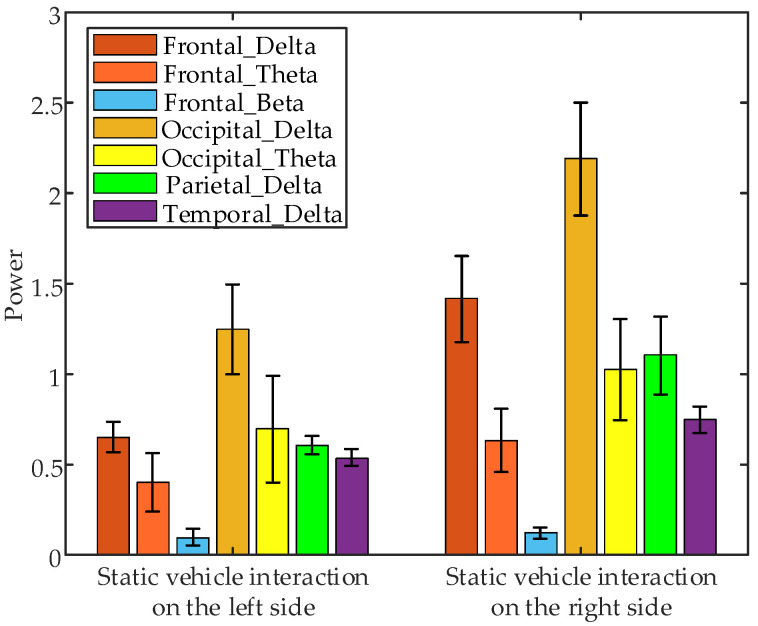
Error bar plots of the average EEG parameters of all subjects during the 1 s interaction under different interaction positions.

Non-parametric tests conducted on EEG data collected during the 1 s interaction at different positions revealed significant differences in the following EEG parameters: frontal δ band power (*p* = 0.0495 < 0.05), frontal θ band power (*p* = 0.0433 < 0.05), frontal β band power (*p* = 0.0045 < 0.05), occipital δ band power (*p* = 0.0495 < 0.05), occipital θ band power (*p* = 0.0433 < 0.05), parietal δ band power (*p* = 0.0495 < 0.05), and temporal δ band power (*p* = 0.0495 < 0.05) between interaction with static vehicle on the left and right sides. Figure 20 shows the error bar plots of the average EEG parameters of all subjects during the 1 s interaction at different positions. The results indicate that the power of significant EEG features during interactions with static vehicles on the right side is notably higher compared to those on the left side.

**Figure 21:**
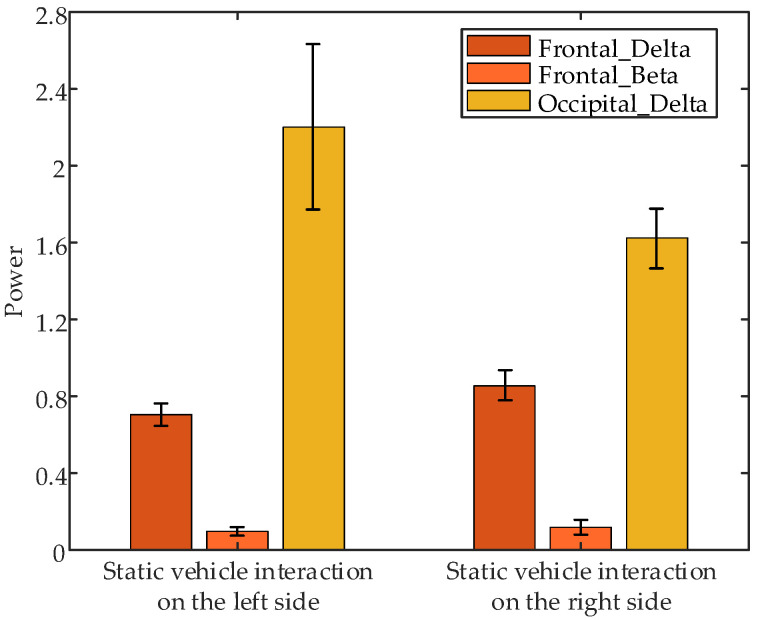
Error bar plots of the average EEG parameters of all subjects during the 1 s after interaction under different interaction positions.

Non-parametric tests on EEG data collected during the 1 s after interaction at different positions revealed significant differences in the following parameters: frontal δ band power (*p* = 0.0495 < 0.05), frontal β band power (*p* = 0.0167 < 0.05), and occipital δ band power (*p* = 0.0495 < 0.05) between interaction with static vehicles on the left and right sides. Figure 21 presents the error bar plots of the average EEG parameters of all subjects during the 1 s after an interaction at different positions. The results indicate that occipital δ band power is highest during interactions with static vehicles on the left side, while frontal δ and β band powers are highest during interactions with static vehicles on the right side.

Based on the comprehensive analysis, significant differences in EEG signal characteristics are observed when stimulated by different interaction positions during the 1 s before the interaction and the 1 s after the interaction. These differences are less pronounced during the 1 s after interaction. Notable differences before and during the interaction mainly occur in the frontal, occipital, parietal, and temporal lobes, whereas after the interaction, differences are primarily observed in the frontal and occipital lobes. Increased power values in various brain regions during interactions with the stationary vehicle on the right side suggest more concentrated and active neural activity when processing the vehicle parked on the right side. Video observations indicate that interactions with the stationary vehicle on the right side occur closer to the vehicle compared to those on the left side, which might contribute to this phenomenon.

### 3.5. Different Interaction Brightness Stimulus Correlation Analysis

**Figure 22:**
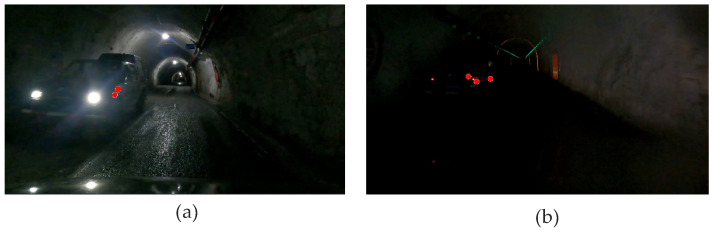
Diagram of static vehicle interaction in different brightness. (**a**) Interaction with a dynamic vehicle in a bright environment; (**b**) interaction with a dynamic vehicle in a dark environment.

To analyze the effect of varying interaction brightness on subject EEG signals, EEG data from interactions with a dynamic vehicle in the tunnel of different brightness levels were selected for analysis. For each signal, EEG data were segmented into three intervals: 1 s before interaction, 1 s during interaction, and 1 s after interaction. Feature selection followed the procedures outlined in Section 3.3. The different levels of interaction brightness are illustrated in Figure 22.

**Figure 23:**
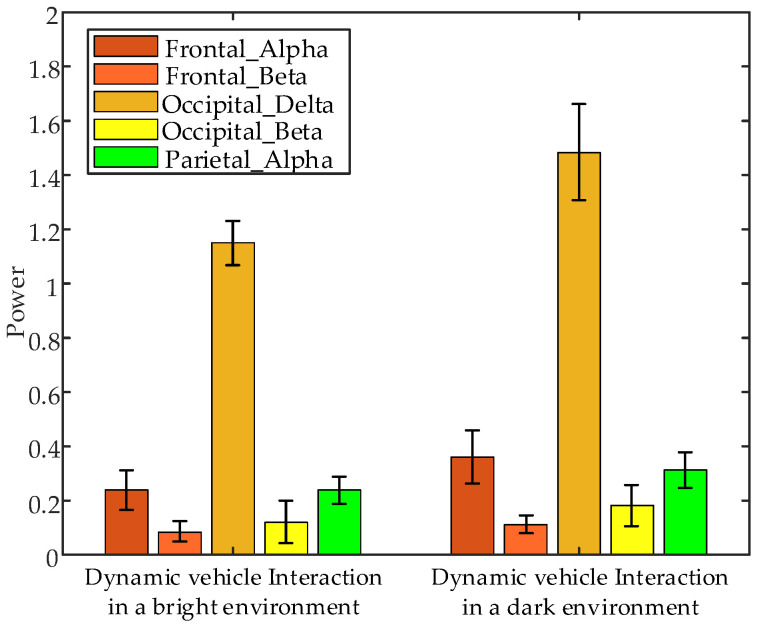
Error bar plots of the average EEG parameters of all subjects during the 1 s before interaction under different interaction brightness.

Non-parametric tests performed on EEG data from the 1 s before interaction under various brightness levels revealed significant differences in the following parameters: frontal α band power (*p* = 0.0374 < 0.05), frontal β band power (*p* = 0.0062 < 0.05), occipital δ band power (*p* = 0.0495 < 0.05), occipital β band power (*p* = 0.0069 < 0.05), and parietal α band power (*p* = 0.0250 < 0.05) between interaction with dynamic vehicle in bright and dark environments. The error bar plots of the average EEG parameters of all subjects during the 1 s before interaction under different brightness conditions are displayed in Figure 23. These results indicate that feature power values are significantly higher during interactions with a dynamic vehicle in dark environments compared to bright environments.

**Figure 24:**
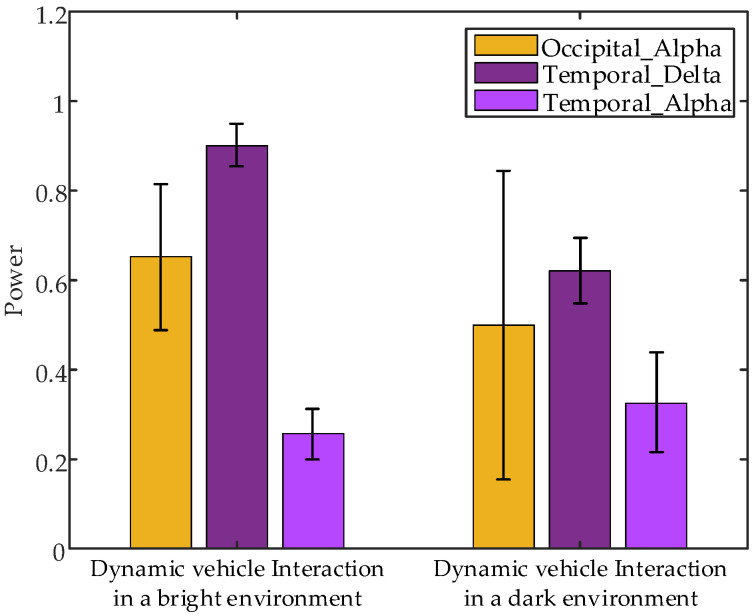
Error bar plots of the average EEG parameters of all subjects during the 1 s interaction under different interaction brightness.

Non-parametric tests on EEG data from the 1 s interaction under various brightness levels revealed significant differences in the following parameters: occipital α band power (*p* = 0.0374 < 0.05), temporal δ band power (*p* = 0.0495 < 0.05), and temporal α band power (*p* = 0.0163 < 0.05) between interaction with dynamic vehicles in bright and dark environments. Figure 24 shows the error bar plots of the average EEG parameters of all subjects during the 1 s interaction under different brightness conditions. The results indicate that occipital α band power and temporal δ band power are higher during interactions with dynamic vehicles in bright environments compared to dark environments, whereas temporal α band power is lower in bright environments.

**Figure 25:**
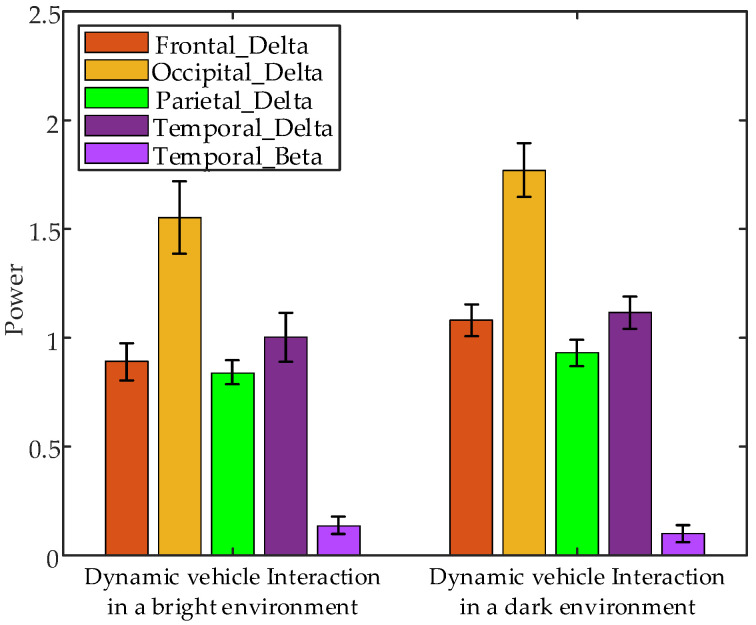
Error bar plots of the average EEG parameters of all subjects during the 1 s after interaction under different interaction brightness.

Non-parametric tests on EEG data from the 1 s after interaction, under different brightness levels, revealed significant differences in the following parameters: frontal δ band power (*p* = 0.0495 < 0.05), occipital δ band power (*p* = 0.0495 < 0.05), parietal δ band power (*p* = 0.0495 < 0.05), temporal δ band power (*p* = 0.0495 < 0.05), and parietal β band power (*p* = 0.0010 < 0.05) between interaction with dynamic vehicle in bright and dark environments. Figure 25 presents the error bar plots of the average EEG parameters of all subjects during the 1 s after interaction under different brightness conditions. The results suggest that temporal β band power is higher during interactions with dynamic vehicles in bright environments compared to dark environments, while the power values of other significant features are lower in bright environments compared to dark environments.

The comprehensive analysis revealed significant differences in EEG signal characteristics under various interaction brightness levels during the 1 s before and 1 s after the interaction, with fewer significant differences observed during the 1 s interaction itself. Specifically, significant differences before interaction occurred primarily in the frontal, occipital, and parietal lobes; during interaction, they were mainly in the occipital and temporal lobes, and after interaction, they were observed predominantly in the frontal, parietal, and occipital lobes. Higher power values in several brain regions before and after interaction with a dynamic vehicle in a dark environment indicate increased neural activity related to observing and processing environmental details under low-light conditions, potentially reflecting heightened visual attention demands. Conversely, during the 1 s interaction, a brief relaxation phase might occur when the interaction is successful, allowing for temporary ease.

## 4. Conclusions

This study investigates the cognitive processes of drivers exposed to various interactive stimuli involving mine vehicles. Simulation experiments were conducted to analyze the effects of different interactions, including dynamic vehicle interaction, static vehicle interaction, personnel interaction, and warning sign interaction, on the EEG signals of the driver. Active brain regions were examined in the time domain and frequency domain, and feature indices of EEG signals from 21 subjects were analyzed under different interaction conditions. Key time points in EEG signals during various interaction phases were identified. Variance analysis and comparisons were performed to assess changes in Delta, Theta, Alpha, and Beta band energies across different interaction phases, providing insights into brain activity state changes under diverse stimuli. Additionally, the average power of each frequency band was mapped onto the scalp in 0.5 s intervals to further investigate brain activity across different regions. Finally, non-parametric tests were applied to EEG signals from different interaction types, positional stimuli, and brightness conditions to analyze variations in brain activity states.

Future research should examine how the responses of drivers vary according to different characteristics and integrate physiological data with behavioral performance to better understand the relationship between cognitive load and safety behavior. This approach could provide a scientific basis for optimizing mine transport vehicle interactions. Additionally, incorporating a range of physiological signals would enable a thorough assessment of the cognitive and response state drivers, leading to a more complete understanding of safety and efficiency in coal mine environments.

## Figures and Tables

**Table 1:** Non-parametric test analysis of the average power of all subjects’ frequency bands grouped over time during the dynamic vehicle interaction process.

Frequency Band	Degrees of Freedom	Chi-Square	*p*
Delta	29	91.1608	<0.001
Theta	29	72.7561	<0.001
Alpha	29	163.0815	<0.001
Beta	29	49.1810	<0.05

## Data Availability

The data presented in this study are available upon request from the corresponding author.
